# A MRI radiomics-based model for prediction of pelvic lymph node metastasis in cervical cancer

**DOI:** 10.1186/s12957-024-03333-5

**Published:** 2024-02-17

**Authors:** Tao Wang, Yan-Yu Li, Nan-Nan Ma, Pei-An Wang, Bei Zhang

**Affiliations:** 1 Suzhou Medical College of Soochow University, Suzhou, China; 2 Department of Radiology, Xuzhou Central Hospital, Xuzhou, China; 3 Department of Gynaecology and Obstetrics, Xuzhou Central Hospital, Xuzhou, China; 4 Hospital Administration Office, Xuzhou Central Hospital, Xuzhou, China

**Keywords:** MRI, Radiomics, Lymph node, Cervical cancer

## Abstract

**Background:**

Cervical cancer (CC) is a common malignancy of the female reproductive tract, and preoperative prediction of lymph node metastasis (LNM) is essential. This study aims to design and validate a magnetic resonance imaging (MRI) radiomics-based predictive model capable of detecting LNM in patients diagnosed with CC.

**Methods:**

This retrospective analysis incorporated 86 and 38 CC patients into the training and testing groups, respectively. Radiomics features were extracted from MRI T2WI, T2WI-SPAIR, and axial apparent diffusion coefficient (ADC) sequences. Selected features identified in the training group were then used to construct a radiomics scoring model, with relevant LNM-related risk factors having been identified through univariate and multivariate logistic regression analyses. The resultant predictive model was then validated in the testing cohort.

**Results:**

In total, 16 features were selected for the construction of a radiomics scoring model. LNM-related risk factors included worse differentiation (*P* < 0.001), more advanced International Federation of Gynecology and Obstetrics (FIGO) stages (*P* = 0.03), and a higher radiomics score from the combined MRI sequences (*P* = 0.01). The equation for the predictive model was as follows: −0.0493–2.1410 × differentiation level + 7.7203 × radiomics score of combined sequences + 1.6752 × FIGO stage. The respective area under the curve (AUC) values for the T2WI radiomics score, T2WI-SPAIR radiomics score, ADC radiomics score, combined sequence radiomics score, and predictive model were 0.656, 0.664, 0.658, 0.835, and 0.923 in the training cohort, while these corresponding AUC values were 0.643, 0.525, 0.513, 0.826, and 0.82 in the testing cohort.

**Conclusions:**

This MRI radiomics-based model exhibited favorable accuracy when used to predict LNM in patients with CC. Relative to the use of any individual MRI sequence-based radiomics score, this predictive model yielded superior diagnostic accuracy.

**Supplementary Information:**

The online version contains supplementary material available at 10.1186/s12957-024-03333-5.

## Introduction

Cervical cancer (CC) is a common malignancy of the female reproductive tract [1]. In CC patients, disease progression is closely related to pelvic lymph node (LN) metastasis (LNM) status [2–4], as early-stage CC patients with and without pelvic LNM exhibit respective 5-year survival rates of 65% and 90% [5]. Postoperative radiotherapy is the most common recommendation for treating CC patients whose postoperative pathological findings reveal evidence of pelvic LNM [6], underscoring the need to accurately judge the pelvic LNM status of these patients so that staging can be performed accurately, prognostic estimates can be generated, and treatment strategies can be planned [7]. Currently, clinical efforts to diagnose pelvic LNM are primarily based on LN morphological characteristics derived from magnetic resonance imaging (MRI), with LN short-axial diameter measurements being the most frequently used in this context [8]. However, this strategy yields relatively low sensitivity rates of 30.3–72.9% when attempting to differentiate between metastatic and non-metastatic LNs [9, 10]. When the biopsy of sentinel LNs can yield a high degree of accuracy and sensitivity, it is an invasive strategy, and the resultant data can be impacted by the skill level of the clinician [11]. It thus remains highly challenging to effectively predict LNM status in CC patients prior to surgery.

Radiomics methods entail the extraction of high-dimensional quantitative data from clinical images, providing a means of characterizing microscopic features in tumors or other tissues not visible to the naked eye [12]. Radiomics strategies have increasingly been used to enhance diagnostic accuracy and prognostic predictive efforts for a range of tumor types [13–15]. Several reports have also demonstrated the utility of radiomics-based methods as a means of enhancing the accuracy of efforts to predict LNM [16–18]. However, there remains a lack of any MRI radiomics-based studies specifically focused on predicting the pelvin LNm status of CC patients.

The present study was thus developed with the goal of establishing and validating an MRI radiomics-based predictive model capable of assessing the LNM status of CC patients.

## Materials and methods

### Study design and patients

The present retrospective analysis received approval from the hospital Institutional Review Board, which waived the requirement for informed consent. This study included a training cohort composed of 86 consecutive CC patients who were evaluated from June 2016 to June 2021, and a testing cohort composed of 38 consecutive CC patients who were evaluated from July 2021 to October 2022.

Patients eligible for inclusion were as follows: (a) CC patients that had hysteroscope-confirmed diagnoses prior to surgery;, (b) CC patients who underwent conventional MRI and diffusion-weighted imaging (DWI) tests within 7 days prior to surgery, and (c) patients who had undergone pelvic LN dissection. Patients were excluded if they are as follows: (a) exhibited incomplete clinical data or (b) had undergone radiotherapy or chemotherapy prior to surgery. The patients in training and test cohorts were included using the same inclusion and exclusion criteria.

For all study participants, the baseline data and MRI-based radiomics data were collected. The baseline data included age, body mass index (BMI), tumor differentiation, depth of tumor invasion, clinical staging according to the 2018 International Federation of Gynecology and Obstetrics (FIGO) criteria, and serum levels of tumor biomarkers including squamous cell carcinoma antigen (SCC), carbohydrate antigen 199 (CA199), alpha-fetoprotein (AFP), and carcinoembryonic antigen (CEA). The MRI-based radiomics data were extracted from T2WI, fat suppression T2WI, and apparent diffusion coefficient (ADC) sequences.

### MRI scanning

A 1.5T MRI instrument (Philips) with a body array coil (Ingenia) was used for all MRI analyses. The scanning sequence for each participant included axial T2WI, axial fat suppression T2WI, axial DWI (*b*-values: 0 and 800 s/mm^2^), sagittal T1WI, and sagittal T2WI. Fat suppression was achieved through a spectral attenuated inversion recovery (SPAIR) sequence. On the axial T2-weighted image, the radiologists delineated regions of interest at all levels of the lesion, and then the software automatically generated the volume of interest (VOIs) and copied them to the ADC map. For detailed information regarding each scanning sequence, see Table 1.

**Table 1:** The parameters of the MRI

	TR/TE (ms)	FOV (mm)	Acquisition matrix	Slice thickness (mm)	Slice gap (mm)	NSA
Sagittal T2WI	3935/80	260 × 300	288 × 250	6	1	2
Axial T2WI-SPAIR	1450/100	210 × 210	252 × 200	3	1	2
Axial DWI	3040/65	375 × 300	124 × 101	6	1	7
Sagittal T1WI	400/10	210 × 210	210 × 165	3	1	1
Axial T2WI	5780/120	200 × 200	250 × 190	5	1	2

*DWI* Diffused-weight imaging, *FOV* Field of view, *NSA* Number of excites, *TE* Echo time, *TR* Repetition time

### Tumor segmentation and feature extraction

**Fig. 1:**
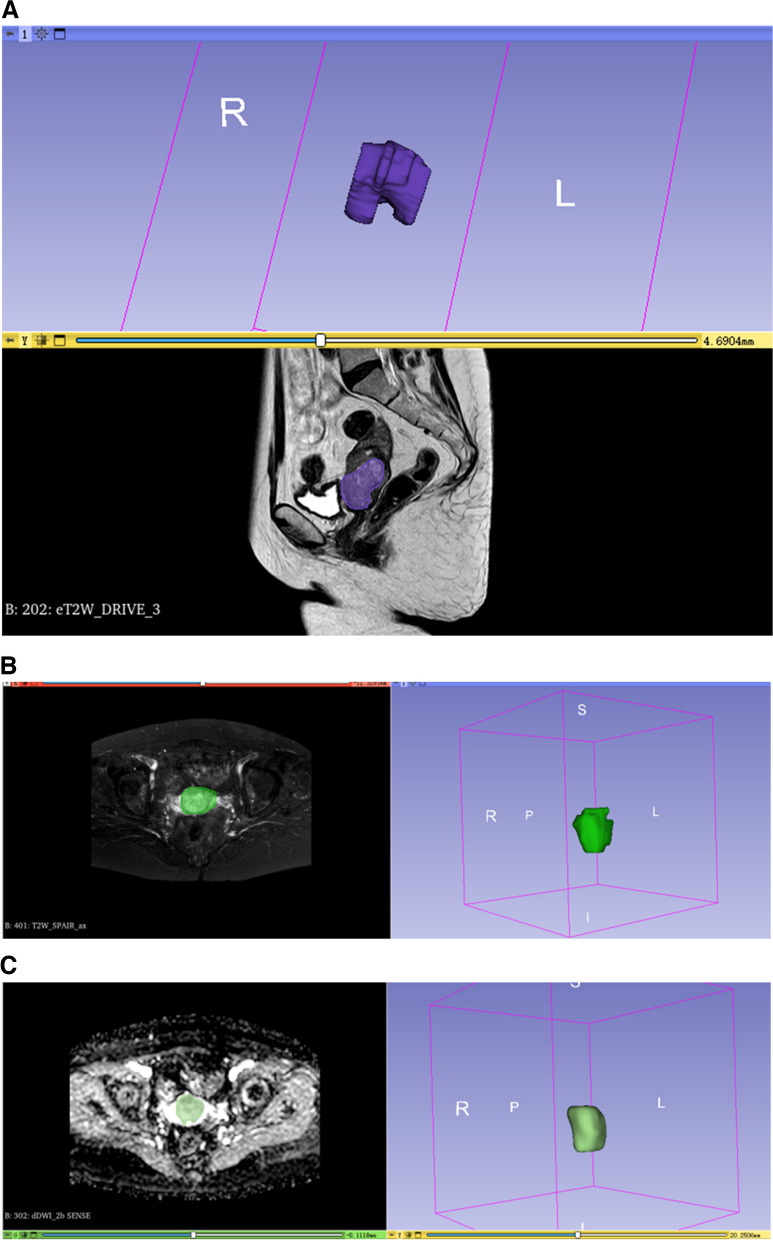
The figures of MRI segmentation on the sequences of **a** T2WI, **b** T2WI-SPAIR, and **c** ADC

Analyses of the sagittal T2WI, axial T2WI-SPAIR, and axial ADC images were conducted, using the 3D Slicer software (v 5.03) to manually draw tumor boundaries to define VOIs. Two radiologists with 5 and 10 years of experience delineated these VOIs while blinded to the pathological results for these patients. For the resultant MRI segmentation, see Fig. 1. The 3D Slicer program was additionally used for feature extraction, and intra- and inter-class coefficient (ICC) values were used to assess observer consistency. MRI images from 20 patients selected at random from the training cohort were independently segmented by the two radiologists for the independent segmentation of target lesions. Reader 1 additionally segmented the tumors from the same 20 patients after a 1-week interval. Only those features exhibiting an *ICC* ≥ 0.9 were regarded as being highly repeatable such that they were retained for further analysis. Reader 1 was then responsible for segmenting all remaining images.

### Feature selection

Feature selection was conducted using a three-step process. Initially, the variance threshold method was used to identify all features with a variance > 0.8 for inclusion in the subsequent step. Then the SelecKBest method was implemented, and all features exhibiting a *P*-value > 0.05 were included in the next step. Lastly, LNM-associated features were selected with a least absolute shrinkage and selection operator (LASSO) regression model. The selected features were used to construct a radiomics signature such that radiomics scores were calculated for all CC patients.

### MRI radiomics-based model establishment and validation

The outcome in this study was LNM status, and the MRI radiomics-based model was established to predict the LNM(+). The LNM status was assessed according to the postoperative pathological results. Univariate and multivariate logistic regression analyses were then used to identify LNM-related risk factors in the training cohort to facilitate the combination of radiomics scores, clinical features, and serum biomarker data. A predictive nomogram was then constructed according to the LNM-related risk factors. Area under the receiver operator characteristic (ROC) curve (AUC) was assessed the accuracy of the predictive model. The data in the test cohort were put into the MRI radiomics-based model to validate the accuracy of the predictive model.

### Benefits of clinical application of predictive model

To assess the clinical utility of the predictive model, decision curve analysis was utilized to evaluate the net benefit of the predictive model in both training and test cohorts.

### Statistical analyses

SPSS 25.0 and R 4.1.2 were used to analyze all data. Categorical data were compared with the *χ*^2^ test of Fisher’s exact test. Continuous data were compared with independent sample *t*-tests and Mann-Whitney *U*-tests when normally and non-normally distributed, respectively. LNM-related risk factors were identified with univariate and multivariate logistic regression analyses. AUC values of the ROC were compared with the DeLong test.

## Results

### Characteristics of the training cohort

The training cohort included 86 CC patients (Table 2), including 64 (74.4%) and 22 (25.6%) without and with LNM, respectively. No significant differences in age, BMI, cancer type, or serum cancer biomarker levels were observed when comparing LNM(−) and LNM(+) patients. However, significant differences between these groups were observed with respect to tumor differentiation, tumor invasion depth, and FIGO staging. Specifically, significantly higher proportions of LNM(+) patients were exhibiting poor tumor differentiation (40.1% vs. 6.2%, *P* < 0.001), cervical stromal invasion depth ≥ 1/2 (68.2% vs. 35.9%, *P* = 0.017), and FIGO stage 3 cancer (9.1% vs. 1.6%, *P* < 0.001) as compared to LNM(−) patients.

**Table 2:** Baseline data of the patients in the training group

	Training (*n* = 86)			Test (*n* = 38)			*p*-value*
	LNM (−)	LNM (+)	*p*-value	LNM (−)	LNM (+)	*p*-value	
Patients’ number	64	22	-	23	15	-	-
Age (y)	56 (Q1: 48; Q3: 60)	56 (Q1: 50; Q3: 60)	0.905	58.6 ± 11.3	52.7 ± 8.6	0.075	0.41
BMI	22.5 ± 2.8	22.8 ± 2.8	0.671	22.9 ± 2.4	21.8 ± 2.2	0.147	0.729
Differentiation			0.001			0.059	0.066
Poor	4 (6.3%)	9 (40.9%)		4 (17.4%)	7 (46.7%)		
Moderate	25 (39.0%)	11 (50%)		11 (47.8%)	7 (47.7%)		
Well	35 (54.7%)	2 (9.1%)		8 (34.8%)	1 (6.6%)		
Cervical stromal invasion depth			0.017			0.335	0.138
< 1/2	41 (64.1%)	7 (31.8%)		11 (47.8%)	4 (26.7%)		
≥ 1/2	23 (35.9%)	15 (68.2%)		12 (52.2%)	11 (73.3%)		
SCC			0.186			0.294	0.782
< 2.5 μg/L	20 (31.3%)	11 (50%)		9 (39.1%)	3 (20%)		
≥ 2.5 μg/L	44 (68.7%)	11 (50%)		14 (60.9%)	12 (80%)		
Ca199			0.748			0.138	0.574
< 37 U/L	54 (84.4%)	18 (81.8%)		19 (82.6%)	15 (100%)		
≥ 37 U/L	10 (15.6%)	4 (18.2%)		4 (17.4%)	0 (0%)		
AFP			0.101			0.063	1.000
< 7 μg/L	50 (78.1%)	21 (95.5%)		17 (73.9%)	15 (100%)		
≥ 7 μg/L	14 (21.9%)	1 (4.5%)		6 (26.1%)	0 (0%)		
CEA			0.759			0.223	1.000
< 5 μg/L	52 (81.3%)	17 (77.3%)		20 (87.0%)	10 (66.7%)		
≥ 5 μg/L	12 (18.7%)	5 (22.7%)		3 (13.0%)	5 (33.3%)		
FIGO stage			0.001			0.591	1.000
I	38 (59.4%)	4 (18.2%)		11 (47.8%)	7 (46.7%)		
II	23 (39.1%)	16 (72.7%)		12 (52.2%)	7 (46.7%)		
III	1 (1.5%)	2 (9.1%)		0 (0%)	1 (6.6%)		
Cancer types			0.843			0.114	0.561
Adenocarcinoma	17 (26.6%)	7 (31.8%)		7 (30.4%)	1 (6.6%)		
Others	47 (74.4%)	15 (68.2%)		16 (69.6%)	14 (93.4)		

*AFP α*-fetoprotein, *BMI* body mass index, *CEA* carcinoembryonic antigen, *FIGO* International Federation of Gynecology and Obstetrics, *SCC* squamous cell carcinoma antigen. *P-values between training and test groups

### Feature selection and radiomics score calculation

In total, 851 radiomics features were extracted per scan sequence (T2WI, T2WI-SPAIR, and ADC). A step-by-step process was then used to select features for these sequences and for a combination of these three scan sequences to facilitate the establishment of a radiomics signature. In total, 16 features were ultimately selected for use when calculating radiomics scores (Supplementary Table 1). Coefficient values for each feature and the mean square error for the combined sequences are presented in Supplementary Figure 1.

### Predictive model establishment

Univariate analyses revealed that worse differentiation (*P* < 0.001), cervical stromal invasion depth ≥ 1/2 (*P* = 0.01), more advanced FIGO stage (*P* < 0.001), and higher combined sequence-based radiomics scores (*P* < 0.001) were all related to CC patient LNM status. In a multivariate analysis, LNM-related risk factors included worse differentiation (*P* < 0.001), more advanced FIGO stage (*P* = 0.03), and higher combined sequence-based radiomics scores (*P* = 0.01, Table 3).

**Table 3:** Risk factors of the LNM

	Univariate analysis			Multivariate analysis		
	OR	95% *CI*	*p*-value	OR	95% *CI*	*p*-value
Age	1	0.96–1.05	0.87			
BMI	1.04	0.87–1.24	0.66			
Differentiation	0.16	0.07–0.4	< 0.001	0.13	0.04–0.42	< 0.001
Cervical stromal invasion depth	3.82	1.36–10.72	0.01	1.68	0.35–7.99	0.51
SCC	0.45	0.17–1.22	0.12			
Ca199	1.2	0.33–4.3	0.78			
AFP	0.17	0.02–1.38	0.1			
CEA	1.27	0.39–4.14	0.69			
FIGO stage	5.31	1.89–14.88	< 0.001	5.59	1.23–25.54	0.03
Cancer types	0.78	0.27–2.23	0.64			
Radiomics score of combined sequences	9398.37	83.65–1055,939.57	< 0.001	1387.69	4.43–434,225.89	0.01

*AFP α*-fetoprotein, *BMI* body mass index, *CEA* carcinoembryonic antigen, *FIGO* International Federation of Gynecology and Obstetrics, *LNM* lymph node metastasis, *SCC* squamous cell carcinoma antigen

**Fig. 2:**
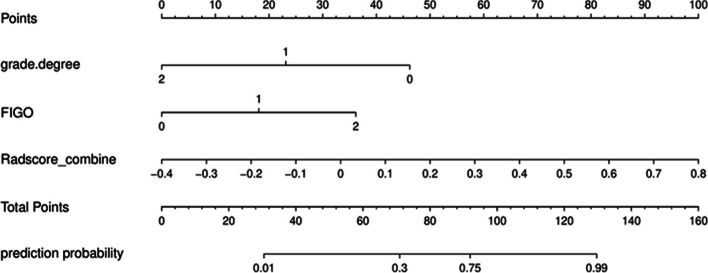
The nomogram of predictive model

These results were next used to construct a predictive model, the nomogram for which is presented in Fig. 2. The formula used to compute nomogram scores for this model was as follows: score = −0.0493–2.1410 × differentiation level (0: poor; 1: moderate; 2: well) + 7.7203 × combined sequence radiomics score + 1.6752 × FIGO stage (0: I; 1: II; 2: III). To maximize sensitivity and specificity, we selected a cut-off score of 0.662 (sensitivity = 81.8%, specificity = 85.9%). If the score was greater than or equal to 0.662, the patient was considered to be LNM(+). If the score was less than 0.662, the patient was considered to be LNM(−).

**Fig. 3:**
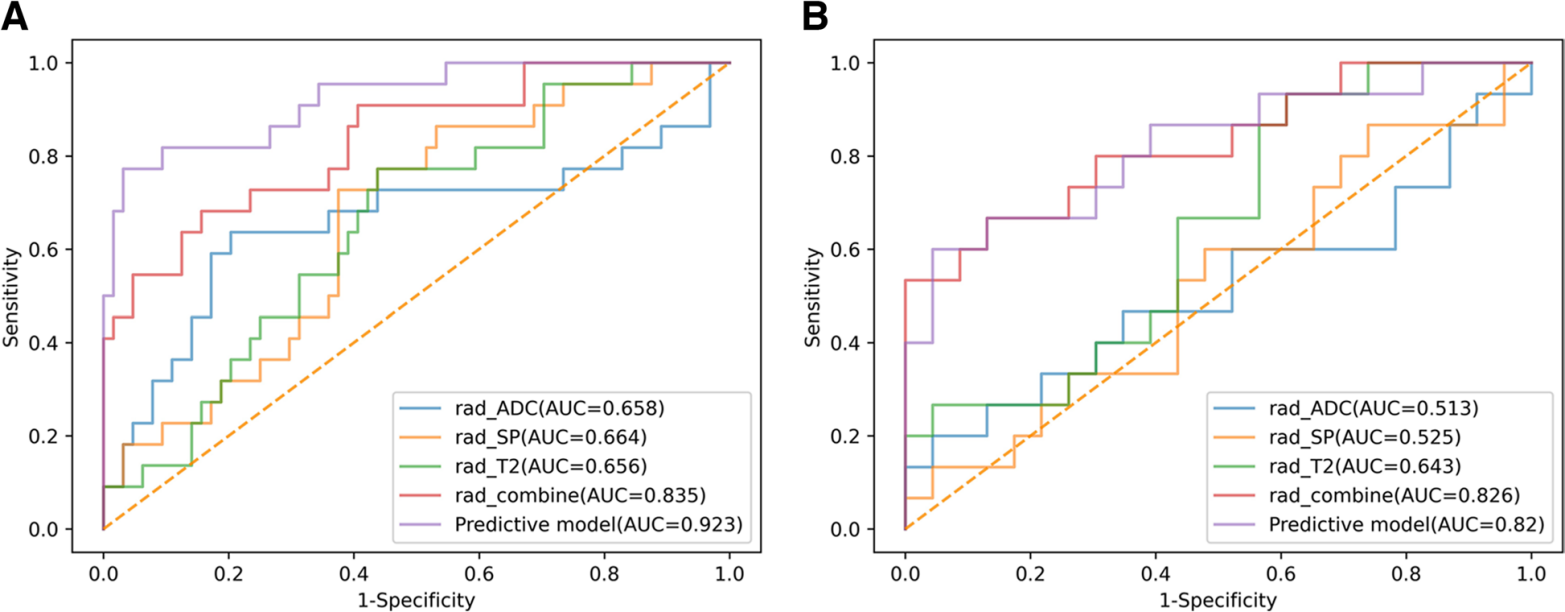
The ROC curves of radiomics score of T2WI, radiomics score of T2WI-SPAIR, radiomics score of ADC, radiomics score of combined sequences, and the predictive model in the **a** training and **b** test groups

The AUC values for the T2WI, T2WI-SPAIR, ADC, and combined sequence radiomics scores, as well as the combined predictive model, were 0.656, 0.664, 0.658, 0.835, and 0.923, respectively (Fig. 3A, Table 4). The AUC for the radiomics score based on the combination of three sequences was significantly larger than the corresponding AUC values for radiomics scores computed based upon the T2WI (*P* = 0.005), T2WI-SPAIR (*P* = 0.008), and ADC (*P* = 0.01) models. The predictive model exhibited a significantly higher AUC value as compared to the combined sequence-based radiomics score (*P* = 0.04).

**Table 4:** Diagnostic performance of each parameter

	Training group			Test group		
	Sensitivity	Specificity	AUC	Sensitivity	Specificity	AUC
Radiomics score of T2WI	68.2%	59.4%	0.656	53.3%	56.5%	0.643
Radiomics score of T2WI-SPAIR	45.5%	64.1%	0.664	33.3%	60.9%	0.525
Radiomics score of ADC	50%	82.8%	0.658	33.3%	78.3%	0.513
Radiomics score of combined sequences	68.2%	78.1%	0.835	60%	87%	0.826
Predictive model in this study	81.8%	85.9%	0.923	66.7%	78.3%	0.82

*ADC* apparent diffusion coefficient, *AUC* area under the curve

### Model validation

The testing group included 38 patients (Table 2), including 23 (72.2%) and 15 (27.8%) without and with LNM, respectively. No significant differences in baseline data were observed when comparing the training and testing cohorts (Table 2). The AUC values for the T2WI, T2WI-SPAIR, ADC, and combined sequence radiomics scores, as well as the combined predictive model, were 0.643, 0.525, 0.513, 0.826, and 0.82, respectively (Fig. 3B, Table 4). The AUC for the radiomics score based on the combination of three sequences was significantly larger than the corresponding AUC values for radiomics scores computed based upon the T2WI (*P* = 0.04), T2WI-SPAIR (*P* = 0.003), and ADC (*P* = 0.002), respectively. The AUC value for the predictive model was similar to that for the radiomics score based on the combined sequences (*P* = 0.94).

### Potential clinical benefits of the predictive model

**Fig. 4:**
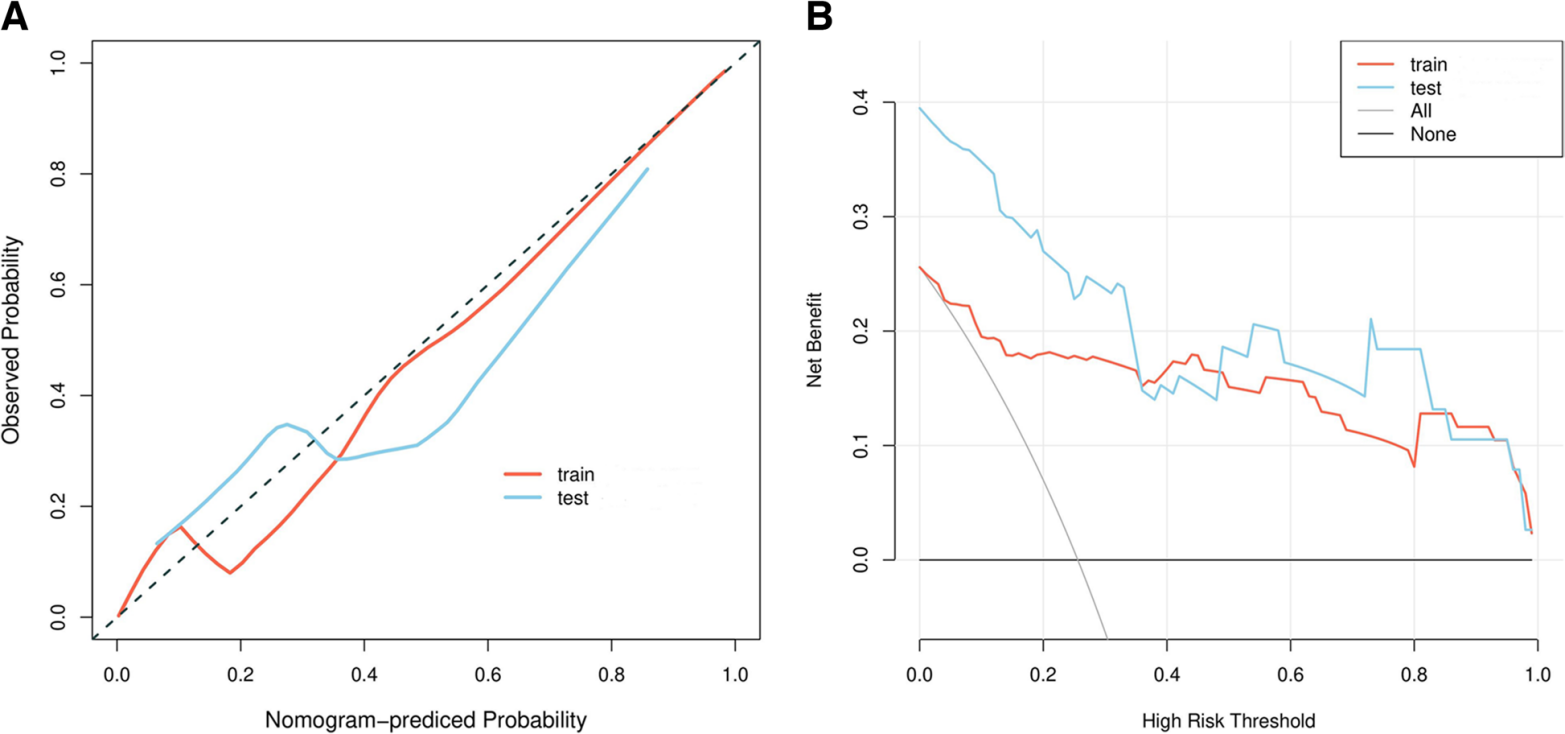
The **a** calibration curves and **b** decision curve analysis of nomograms of predictive model

Calibration curves revealed a high degree of consistency between predicted and actual LNM status when using the predictive model in both the training and testing cohorts (Fig. 4A). Decision curves generated for this nomogram additionally revealed that this predictive model was associated with net benefits in both patient cohorts, with a risk threshold greater than 0 (Fig. 4B).

## Discussion

In patients with CC, the ability to detect LNM prior to surgery is vital for effective treatment planning. Here, MRI-based radiomics score values, FIGO staging, and tumor differentiation were all identified as significant predictors of the LNM status of CC patients. When these factors were combined to develop a predictive model, we can calculate the risk score for each patient. For one, we can compare the patients’ risk score to the cut-off score of the predictive model and predict the probability of the LNM. In addition, we can get the prediction probability directly by referring the nomogram of predictive model according to the patients’ risk score.

Conventional MRI-based approaches to assessing LNM status primarily center on LN sizing, with metastatic LNs being defined as nodes exhibiting a short-axis diameter > 10 mm in most cases [19]. However, this approach yields low sensitivity levels (30–73%) when attempting to differentiate between patients with and without LNM [9, 10]. This is consistent with the observations of Williams et al. [9], who determined that the short-axis diameter of 54.4% of metastatic LNs was less than 10 mm. While Koh et al. [20] suggested respective short-axis diameter thresholds of > 8 mm and > 10 mm when assessing the metastatic status of pelvic and retroperitoneal LNs as a means of improving diagnostic performance, LNs harboring micro-metastases can be normally sized such that they will not be detected through conventional MRI scans.

DWI offers a means of potentially detecting malignancies and metastatic LNs [19]. In their analyses of CC patients, Zhang et al. [19] determined that ADC_mean_ and ADC_min_ were respectively associated with the highest levels of diagnostic accuracy when evaluating enlarged LNs and normally sized LNs, but the corresponding AUC values for these two parameters were just 0.644 and 0.758. This highlights the potential importance of extracting additional information from images in an effort to better assess tumor heterogeneity and to improve diagnostic performance.

For the present analysis, radiomics features were extracted from the T2WI, T2WI-SPAIR, and ADC sequences, all of which are frequently employed when assessing and analyzing CC and LN status. DWI can provide effective insight into tissue movement at the molecular level, yielding information regarding tumor cell infiltration and diffusion. T2WI sequences can highlight tumor morphology and anatomical structures, enabling the quantification of dimensional and morphological parameters that can enable the detection of relatively subtle tumor invasivity. Radiomics scores based on the combined sequence exhibited good predictive performance in both the training and testing cohorts, with respective AUCs of 0.835 and 0.826, both of which were higher than the AUCs associated with radiomics scores derived from individual T2WI, T2WI-SPAIR, and ADC sequences. This suggests that multiple features should be used when extracting radiomics features in order to improve the predictive performance of these features.

In addition to radiomics scores, poorer tumor differentiation and more advanced FIGO staging were both associated with LNM in CC patients. Both of these factors are indicative of higher-grade malignancies, consistent with greater LNM risk. In line with these results, Huang et al. [21] previously reported that worse differentiation was associated with an increase in the risk of LNM in patients with gastric cancer. Similarly, FIGO stage was also found to be strongly linked to the prognosis of the CC patients [22].

The predictive model developed herein exhibited respective AUC values of 0.923 and 0.82 in the training and validation cohorts. Both of these were higher than the respective AUC values of 0.754 and 0.727 reported in a prior study focused on designing a predictive MRI radiomics-based model for LNM diagnosis in CC patients published by Li et al. [23]. While MRI radiomics scores were implemented in both the present study and this past analysis, Li et al. only included red blood cell counts when assessing clinical risk factors [23], whereas tumor differentiation and FIGO staging were both risk factors that were incorporated into the present analyses. This suggests that differentiation and FIGO staging may offer more representative information regarding a target tumor as compared to red blood cell counts.

In the training cohort, the AUC for the predictive model was significantly higher than that for the combined sequence-based radiomics score alone (0.923 vs. 0.835, *P* = 0.04). This suggests that the incorporation of clinical data may further improve the diagnostic utility of radiomics scores. However, AUC values did not differ significantly in the testing cohort (0.82 vs. 0.826, *P* = 0.94). This is likely because there were no significant differences in tumor differentiation and FIGO staging between patients in this cohort with and without LNM.

There are some limitations to these analyses. For one, the retrospective nature of these analyses renders them highly susceptible to potential bias. In addition, this was a single-center study. Additional prospective multicenter validation will thus be essential. Moreover, measurement errors cannot be entirely avoided when manually defining lesion boundaries, and factors such as edema, hemorrhage, necrosis, and degeneration can contribute to such errors. Third, radiomics strategies are often limited in their reproducibility and amenability to standardization, potentially restricting their utility [24].

## Conclusions

In summary, the MRI radiomics-based model developed herein exhibited great promise as an accurate tool for predicting the LNM status of patients with CC. Relative to the use of radiomics scores based on any one MRI sequence, the combined predictive model established in this study was associated with significant improvements in overall diagnostic accuracy.

### Supplementary Information

**Additional file 1:** **Supplementary Figure 1.** The (a) coefficient of each feature and (b) mean square error of the combined sequences.

**Additional file 2:** **Supplement Table 1.** Radiomics features and their coefficients.

## Data Availability

The data that support the findings of this study are available from the corresponding author upon reasonable request.

## Abbreviations

ADC: Apparent diffusion coefficient
AFP: Alpha-fetoprotein
AUC: Area under curve
CA199: Carbohydrate antigen 199
CC: Cervical cancer
CEA: Carcinoembryonic antigen
DWI: Diffused weighted imaging
FIGO: International Federation of Gynecology and Obstetrics
LASSO: Least absolute shrinkage and selection operator
LN: Lymph node
LNM: LN metastasis
MRI: Magnetic resonance imaging
ROC: Receiver operator characteristic
SCC: Squamous cell carcinoma antigen
VOI: Volume of interest
