# Supplementary figures and images for: A MRI radiomics-based model for prediction of pelvic lymph node metastasis in cervical cancer
Source: World J Surg Oncol. 2024 Feb 17;22:55. doi: 10.1186/s12957-024-03333-5 (PMC10873981; doi:10.1186/s12957-024-03333-5)

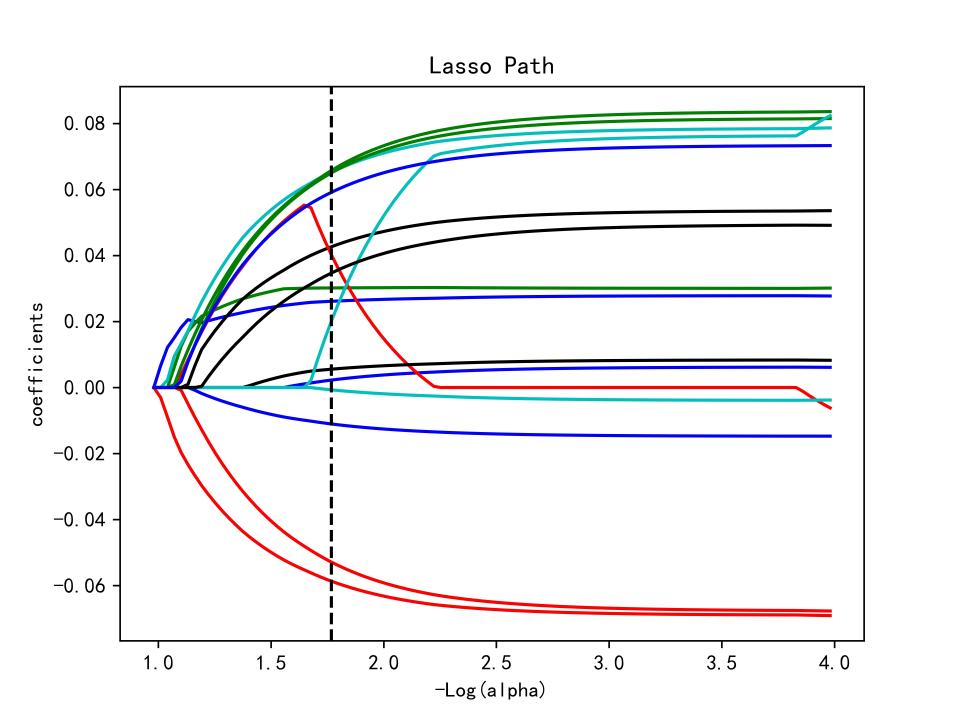

Supplement: Supplementary file 1 — Additional file 1: Supplementary Figure 1. The (a) coefficient of each feature and (b) mean square error of the combined sequences. [file 12957_2024_3333_MOESM1_ESM.zip › supplement Fig 1a.jpg]

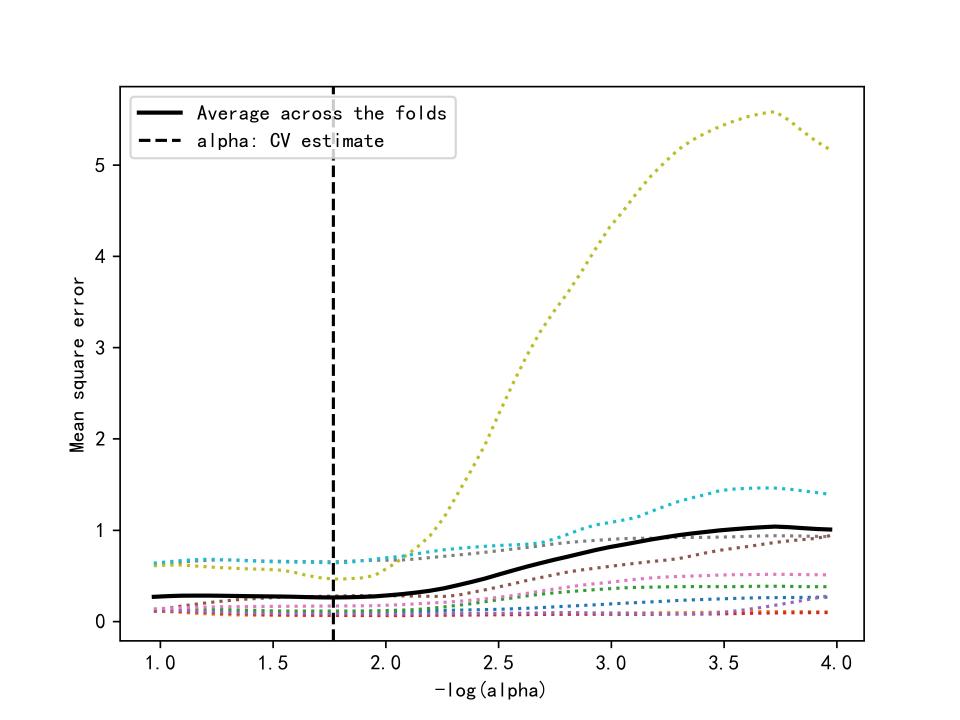

Supplement: Supplementary file 1 — Additional file 1: Supplementary Figure 1. The (a) coefficient of each feature and (b) mean square error of the combined sequences. [file 12957_2024_3333_MOESM1_ESM.zip › supplement Fig 1b.jpg]
