# Supplementary material for: A MRI radiomics-based model for prediction of pelvic lymph node metastasis in cervical cancer
Source: World J Surg Oncol. 2024 Feb 17;22:55. doi: 10.1186/s12957-024-03333-5 (PMC10873981; doi:10.1186/s12957-024-03333-5)
Supplement: Supplementary file 2 — Additional file 2: Supplement Table 1. Radiomics features and their coefficients. [file 12957_2024_3333_MOESM2_ESM.doc]

Supplement Table 1 Radiomics features and their coefficients

| Features | Coefficients |
| --- | --- |
| wavelet-HHH_glrlm_LongRunHighGrayLevelEmphasis_ADC | 0.02612191 |
| wavelet-LHH_glcm_Correlation_ADC | -0.058665078 |
| wavelet-LHL_ngtdm_Complexity_ADC | 0.030170011 |
| wavelet-HLL_glcm_Correlation_ADC | 0.065219677 |
| wavelet-HHH_glszm_GrayLevelVariance_ADC | 0.042619372 |
| wavelet-LLL_ngtdm_Complexity_ADC | 0.002244711 |
| wavelet-HHH_glszm_LargeAreaEmphasis_ADC | 0.040347517 |
| wavelet-LHH_glrlm_LongRunLowGrayLevelEmphasis_SP | 0.065719335 |
| wavelet-HHH_gldm_SmallDependenceLowGrayLevelEmphasis_T2 | -0.00069834 |
| original_shape_Flatness_T2 | 0.00554405 |
| wavelet-HLH_glcm_ClusterShade_ADC | -0.011037464 |
| wavelet-LHL_glcm_MCC_SP | -0.052866134 |
| wavelet-LHH_glcm_ClusterShade_ADC | 0.064989288 |
| wavelet-HHL_glszm_LargeAreaEmphasis_ADC | 0.019938118 |
| wavelet-HLL_glcm_ClusterTendency_ADC | 0.034650062 |
| wavelet-LHH_ngtdm_Complexity_T2 | 0.059108834 |
